# Poly(ADP-ribose) polymerase inhibition with HYDAMTIQ reduces allergen-induced asthma-like reaction, bronchial hyper-reactivity and airway remodelling

**DOI:** 10.1111/jcmm.12197

**Published:** 2014-01-20

**Authors:** Laura Lucarini, Alessandro Pini, Elisabetta Gerace, Roberto Pellicciari, Emanuela Masini, Flavio Moroni

**Affiliations:** a Department of NEUROFARBA, Section of Pharmacology and Toxicology, University of FlorenceFlorence, Italy; b Department of Experimental and Clinical Medicine, Section of Anatomy and Histology, University of FlorenceFlorence, Italy; c Department of Chemistry and Drug Technology, University of PerugiaPerugia, Italy

**Keywords:** asthma-like reaction, poly (ADP-ribose) polymerase (PARP), airway inflammation and remodelling, hydroxyl-dimethylaminomethyl-thieno[2,3-c]isoquinolin-5(4*H*)-one (HYDAMTIQ), histamine release

## Abstract

Activation of poly(ADP-ribose) polymerases (PARPs) is considered a key event in the molecular and cellular processes leading from acute asthma attacks to bronchial hyper-reactivity, leucocyte recruitment, chronic inflammation, airway remodelling and lung damage. The present investigation has been carried out to investigate the action of hydroxyl-dimethylaminomethyl-thieno[2,3-c]isoquinolin-5(4*H*)-one (HYDAMTIQ), a new potent PARP inhibitor, in the process leading from asthma-like events to airway damage. Ovalbumin-sensitized guinea pigs exposed two times to allergen inhalation were treated for 8 days with vehicle or HYDAMTIQ. Asthma-like signs, bronchial hyper-reactivity to methacholine, cytokine production, histamine release from mast cells, airway remodelling, collagen deposition and lung damage were evaluated. Repeated HYDAMTIQ administration (1-10 mg/kg/day i.p.) reduced lung PARP activity, delayed the appearance and reduced the severity of allergen-induced cough and dyspnoea and dampened the increased bronchial responses to methacholine. HYDAMTIQ-treated animals presented reduced bronchial or alveolar abnormalities, lower number of eosinophils and other leucocytes in the lung and decreased smooth muscle or goblet cell hyperplasia. The treatment also reduced lung oxidative stress markers, such as malondialdehyde or 8-hydroxy-2′-deoxyguanosine and the lung content of pro-inflammatory cytokines (TNF-α, interleukin (IL)-1β, IL-5, IL-6 and IL-18). Finally, mast cells isolated from the peritoneal or pleural cavities of sensitized, HYDAMTIQ-treated animals had a reduced ability to release histamine when exposed to ovalbumin *in vitro*. Our findings support the proposal that PARP inhibitors could have a therapeutic potential to reduce chronic lung inflammation, airway damage and remodelling in severe unresponsive asthmatic patients.

## Introduction

Asthma is a common airway disorder characterized by recurrent exacerbations of respiratory signs and symptoms (cough, wheezing and dyspnoea), chronic airway inflammation, bronchial hyper-reactivity, mucus plug formation, swelling of air spaces and airway remodelling [1]. Allergens, environmental pollutants, tobacco, infections, specific drugs may trigger asthma attacks in susceptible individuals. An increased number of inflammatory cells such as activated dendritic cells (DC), eosinophils, mast cells, macrophages, T lymphocytes and tissue remodelling processes have been described in the lungs of asthmatic patients and in the airway of sensitized animals used as experimental models of the disease [2, 3].

In spite of the important accomplishments made in the last 20 years in reducing asthma mortality and morbidity, a significant number of patients still have poor responses to available therapies and innovative therapeutic approaches are definitely needed [4, 5]. We previously observed that non-selective poly(ADP-ribose) polymerase (PARPs) inhibitors may significantly reduce the allergen-induced asthma-like reaction in sensitized guinea pigs [6] and comparable results have been independently described in murine asthma models in other laboratories [7, 8]. Poly(ADP-ribose) polymerase are a family of NAD-dependent enzymes able to catalyse the transfer of ADP-ribose units from NAD to substrate proteins [9]. They are particularly abundant in cell nuclei and are involved in the maintenance of genomic integrity, epigenetic regulation of gene expression, control of cell cycle and cell death [10]. Gene targeting approaches associated with the use of small molecular inhibitors showed that at least two of the eighteen members of the PARP family (PARP-1 and PARP-14) are involved in regulating asthmatic airway inflammation. While PARP-14 has only recently been demonstrated to facilitate the differentiation of T cells towards the Th2 phenotype and allergic airways diseases [11], PARP-1, an abundant nuclear protein, has been known for years to contribute to asthmatic airway inflammation possibly because it may affect the function and survival of most of the different cell types present in lungs [7, 8, 12, 13]. Repeated asthmatic episodes cause local oxidative stress with excessive formation of free radicals and damage to endothelia, smooth muscle cells and various lung-resident or immigrating inflammatory cells [13]. This may indeed cause DNA damage with excessive PARP-1 activation, NAD and ATP depletion and cell death because of metabolic derangement [14]. In most cell types present in the lungs, cell death also occurs because excessive PARP-1 activation leads to apoptosis-inducing factor (AIF) release from the mitochondria and subsequent caspase-independent apoptosis [15]. It has also been repeatedly shown that PARP-1 activation may facilitate the expression of tumour necrosis factors (TNF) and other pro-inflammatory cytokines [7, 16–19] and that PARP-1 inhibitors reduce the expression of these genes and eosinophil recruitment in asthmatic lungs [20]. In smooth muscle cells or in fibroblasts exposed to *Escherichia coli* lipopolysaccharide (LPS), PARP-1 silencing and PARP inhibitors have been described to reduce NF-kB (p65) nuclear export and facilitate the expression of iNOS and ICAM-1 [21]. Finally, PARP-1 plays a key role in the maturation of DCs, in the expression of co-stimulatory molecules (CD83 and CD86) and secretion of cytokines [22, 23].

In the present study, we used HYDAMTIQ, a potent and selective PARP-1 inhibitor (IC_50_ of 2–20 nM), previously evaluated in stroke-induced inflammation and brain damage [24] and we tested its action in ovalbumin (OVA)-sensitized guinea pigs exposed to antigen inhalation for two times in 25 days. This approach has been largely used to reproduce the different syndromes of human asthma and to test potential therapeutic agents [13, 25–27]. When repeatedly administered to sensitized guinea pigs, starting a few days after the first antigen challenge, HYDAMTIQ reduced the signs and symptoms of acute asthma-like attack caused by the second prolonged antigen exposure, the subsequent bronchial hyper-reactivity to methacholine (MeCh) and the resulting airway damage, inflammation and remodelling.

## Materials and methods

### Animals

Hartley albino guinea pigs (Rodentia, Bergamo, Italy) were used. Animals were housed in a controlled environment at 22°C with 12 hrs light and had standard chow and water *ad libitum*. The experimental protocol was designed in compliance with the recommendations of the European Community (D.M.116192; O.J. of E.C. L358/1 12/18/1986) for the care and use of laboratory animals. The protocols were approved by the animal care Committee of the University of Florence (Italy).

### Animal sensitization and treatment

The guinea pigs were randomly divided into six experimental groups:

Group 1: Five animals were injected with PBS (5 ml/kg b.wt., i.p., plus 5 ml/kg b.wt. s.c.) and 18 days later received an aerosol of OVA (Fluka, Buchs, Switzerland) dissolved in saline (5 mg/ml). They are referred to as naive OVA-challenged animals.

All the other five groups contained animals sensitized with 100 mg/kg b.wt., i.p., plus 100 mg/kg b.wt. s.c OVA dissolved in saline (20 mg/ml). After 15 days, they were challenged with an OVA aerosol (5 mg/ml saline). Ovalbumin-sensitization was verified by the appearance of cough, as previously reported [6, 13, 25, 26, 28]. By this protocol, only one of 45 animals failed to develop sensitization. Three days later, the sensitized guinea pigs were divided into following groups.

Group 2: Five animals were treated with an intraperitoneal injection (0.5 ml) of vehicle (PBS plus 5% DMSO) for 4 days (18–21) before undergoing the provocation test with an aerosol of saline alone. These are referred to as the sensitized, unchallenged group (sensitized).

Group 3: Five animals were treated with an intra-peritoneal injection (0.5 ml) of PBS for 4 days before undergoing the provocation test with aerosolized OVA (5 mg/ml saline). These are referred to as the sensitized, OVA-challenged group (vehicle).

Groups 4–6: Thirty animals (10 per group) were treated with two daily intraperitoneal injection of HYDAMTIQ (1, 3 and 10 mg/kg/day) synthesized as previously reported [29] and dissolved in 0.5 ml PBS plus 5% DMSO for 4 days before undergoing the second provocation test with aerosolized OVA and for three further day before the pressure at airway opening (PAO) test with MeCh (200 μg/ml). These are referred to as the sensitized, HYDAMTIQ-treated OVA-challenged group.

### Challenge with inhaled OVA and evaluation of respiratory activity

The guinea pigs were individually placed in an airtight transparent whole-body plethysmographic chamber and the changes in inner pressure in the respiratory chamber were monitored as previously reported [6, 25].

The respiratory activity of the animals subjected to the different treatments was monitored for 10 min. [25]. Cough was assumed as a transient change in the pressure (a rapid inspiration followed by a rapid expiration), whereas dyspnoea as a series of irregular breaths of abnormally elevated frequency (tachypnea) and amplitude or as repeated gasping. Movements of the guinea pigs were visually monitored by two trained observers (E.M. and A.P.) who were blinded to group assignment of the animals. The following parameters were evaluated: (*i*) latency time for the first cough stroke (sec.); (*ii*) cough severity, the product of cough frequency (cough strokes/min.), and mean cough amplitude (excess pressure over the normal breath), and (*iii*) latency time for the onset of dyspnoea (sec.).

### Measurement of bronchial constriction

Pentothal (Abbott, Latina, Italy) anaesthetized guinea pigs from each group were mechanically ventilated by a constant volume method with a tidal volume of 10 ml at a rate of 40 strokes/min. as reported previously [25]. Changes in lung resistance to inflation (PAO), specifically the lateral pressure of the inlet air tube, were registered with a polygraph (Battaglia-Rangoni, Bologna, Italy). Each animal was then exposed to an aerosol of MeCh (200 μg/ml) for 1 min. and changes in inflation pressure, which are directly related to airway resistance, were recorded for 5 min. after the beginning of MeCh aerosol and carried out on at least 40 consecutive tracings of respiratory strokes [25].

### Post-mortem analysis

After the second OVA challenge and PAO determination with MeCh, the animals were killed by an excessive dose of anaesthetic. Before opening the thorax, in a selected number of animals, bronchoalveolar lavage (BAL) fluids were obtained by insertion of a cannula into the trachea and instillation of 10 ml of PBS, pH 7.4. The fluid was collected after three flushes into the bronchial tree, centrifuged at 1100 × g for 30 min. and the pellet was resuspended in 5 ml of PBS. The cell-free supernatant was collected and frozen at −80°C for pro-staglandin D_2_ (PGD_2_) determination. The total number of cells present in BAL fluid of sensitized and OVA-challenged animals was counted by light microscopy after trypan blue staining.

The gross appearance of the lungs, liver and kidneys was examined. No macroscopic alterations of these organs that could be related to a toxic effect of HYDAMTIQ treatment were observed. Lung tissue samples from the middle and the lower lobes were taken from each animal, excluded those subjected to BAL for the morphometrical evaluation, for biochemical and morphological analyses, as described below.

### Histological and morphometrical analysis

Tissue samples were fixed by immersion in Mota fluid, dehydrated in graded ethanol and embedded in paraffin. Sections (5 μm) were stained with (*i*) haematoxylin and eosin to evaluate the alveolar and bronchial luminal areas and the thickness of the bronchial smooth muscle layer. This staining permits to clearly identify the smooth muscle layer, composed by eosinophilic spindle-shaped cells, which completely encircles the lumen of small-and medium-sized bronchi and lies between the mucosa and the alveolar parenchyma. The histological and morphometrical analyses were carried out on at least four animals per group, five sections per animals, counting all the small-and medium-sized bronchi present in each slide. (*ii*) Periodic acid-Schiff (PAS) to assess the goblet cell iperplasia, (*iii*) a modified Azan method for collagen fibres to quantify lung collagen deposition [30], (*iv*) Astra blue to evaluate mast-cell granule secretion. The histological and morphometrical measurements were carried out on digital micrographs of the microscopical fields using the ImageJ 1.33 free-share image analysis software (http://rsb.info.nih.gov/ij). The values were obtained from two different observers (A.P. and E.M.).

### Immunohistochemistry for eosinophilic major basic protein (eMBP)

The immunohistochemistry for eosinophil identification was carried as previously reported [25]. Briefly, the sections were treated with 0.3% (v/v) H_2_O_2_ in 60% (v/v) methanol to quench endogenous peroxidase, permeabilized with 0.1% (w/v) Triton X 100 in PBS for 20 min. and incubated overnight with human monoclonal anti-eMBP (clone BMK13; Chemicon, Temecula, CA, USA; working dilution: 1:50 in PBS). Immune reaction was revealed by indirect immunoperoxidase method (Vectastain Elite kit; Vector, Burlingame, CA, USA), using 3,3′-diaminobenzidine as chromogen. In each guinea pig, the number of MBP-positive eosinophils was counted in 10 randomly chosen microscopical fields (test area: 72,346 μm^2^).

### Determination of myeloperoxidase activity

Myeloperoxidase (MPO) activity expressed in mU/mg protein is an indicator of leucocyte accumulation and was determined as previously described [31].

### Determination of 8-OH-2′-deoxyguanosine

DNA was purified [28] and filtered with an Amicon Micropure-EZ filter (Millipore Corporation, Billerica, MA, USA) and 50 μl of each sample was used for 8-OH-2-deoxyguanosine (8-OHdG) determination using a Bioxytech enzyme immunoassay kit (Oxis, Portland, OR, USA), following manufacturer instructions.

### Determination of malondialdehyde

Malondialdehyde (MDA) was determined by measurement of the chromogen obtained from the reaction of MDA with 2-thiobarbituric acid as reported previously [25, 32]. The values are expressed as nM of thiobarbituric acid reactive substances (MDA equivalents)/mg of protein, using a standard curve of 1,1,3,3-tetramethoxypropane.

### Measurement of Manganese superoxide dismutase (MnSOD) activity

The frozen lung samples were homogenized with 10 mM PBS, pH 7.4, sonicated on ice for 1 min., and centrifuged at 100 × g for 10 min. Supernatants were used for SOD measurement. The assay of MnSOD activity was carried out based on SOD-induced inhibition of the conversion of nitro blue tetrazolium (NBT) into formazan mediated by O_2_^−^ generated by xanthine-xanthine oxidase. The reaction was performed in sodium carbonate buffer, 50 mM, pH 10.1, containing 0.1 mM EDTA, 25 μM NBT (Sigma-Aldrich, Milan, Italy), 0.1 mM xanthine and 2 nM xanthine oxidase (Sigma-Aldrich) as previously reported [33]. The rate of reduction in NBT was monitored with a spectrophotometer (Lambda 5; Perkin Elmer, Monza (MB), Italy) set at 560 nm. The amount required to inhibit the rate of reduction in NBT to formazan by 50% was defined as one unit of SOD activity. Specific MnSOD activity was calculated by inhibiting total SOD activity pre-incubating the sample for 30 min. with 2 mM NaCN. Values are mU/mg of protein.

### Western blotting analysis

Lung tissues or BAL cell pellets were homogenized in 700 μl of RIPA buffer plus protease inhibitors and centrifuged at 12,000 × g for 5 min. The supernatant was transferred in tube and total protein levels were quantified using Protein Assay of Pierce (Rockford, IL, USA). Western blotting was determined using a mouse monoclonal anti-PAR(10H) antibody (Alexis Biochemicals, Vinci, FI, Italy) diluted 1:1000 in TBS-T containing either 5% non-fat dry milk (anti-PAR) as previously reported [34]. This monoclonal antibody recognizes poly(ADP-ribose) synthesized by PARP enzymes. The QuantityOne analysis software (Bio-Rad, Hercules, CA, USA) was used.

### Histamine release from isolated mast cells

Mast cells were isolated from sensitized guinea pig by peritoneal and pleural washing as previously reported [35, 36]. After isolation, cells were incubated for 30 min. with different concentration of OVA and histamine was measured fluorimetrically in the supernatant and expressed as a percentage of the total cellular content.

### Determination of TNF-α, IL-1β, IL-5, IL-6, IL-18, PgD_2_, in lungs and in BAL fluid

Production of TNF-α and interleukin (IL)-1β, IL-5, IL-6, IL-18 and PgD_2_ were measured using a commercial ELISA kits (Cayman Chemical, Ann Arbor, MI, USA), following the protocol provided by the manufacturer. Results are expressed as ng or pg of substance/μg of protein. Proteins were measured with Bradford method [37].

### Statistical analysis

Data are mean ± SEM. Statistical analysis was performed by one-way anova, followed by Newman–Keuls multiple comparison *post hoc* test. The Graph Pad Prism 5.0 statistical program (GraphPad Software, San Diego, CA, USA) was used.

## Results

### HYDAMTIQ and PARP activity

It has been repeatedly reported that asthma significantly increases PARP activity and PAR content both in lungs and in cells present in BAL [6, 7]. To reduce this activity, we used HYDAMTIQ, a potent inhibitor of PARP-1 with an IC_50_ of ˜20 nM and PARP-2 (IC_50_ 38 nM). The compound is effective in improving the survival of primary cultures of neuronal cells exposed to oxygen and glucose deprivation, in reducing the loss of ATP content of HeLa cell lines exposed to DNA damaging agents and in reducing infarct volumes and inflammatory cells infiltration in brain after stroke [24]. When the compound has been tested against a panel of 62 receptors and enzymes at a concentration of 10 μM (NOVASCREEN) showed an excellent selectivity: besides PARP-1 and PARP-2, the only other target that was marginally affected being the 5HT transporter (IC_50_ 6 μM).

**Figure 1:**
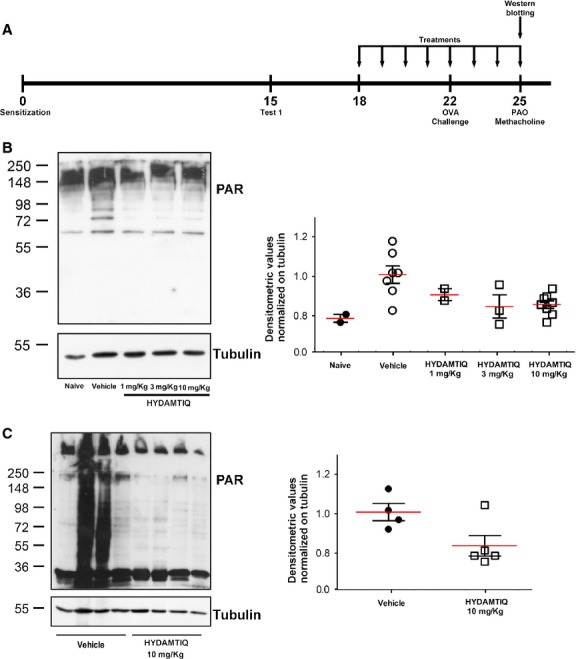
Parylated proteins in guinea pigs lungs and bronchoalveolar lavage (BAL) cells. (A) Experimental protocol. On day 15, ovalbumin sensitization was evaluated with the first antigen challenge. The arrows show the days in which vehicle or HYDAMTIQ treatments were performed. Western blot analysis of PAR expression in lung samples (B) and BAL cells (C). The densitometric analysis was normalized to tubulin and each point report data obtained in a single animal.

Parylated proteins were significantly increased in lung tissue and BAL cells of guinea pigs sensitized, challenged with OVA and sacrificed 3 days later (Fig. 1B and C respectively). HYDAMTIQ treatment significantly reduced PAR content in both specimens (Fig. 1).

### HYDAMTIQ treatment reduced asthma-like reaction

**Figure 2:**
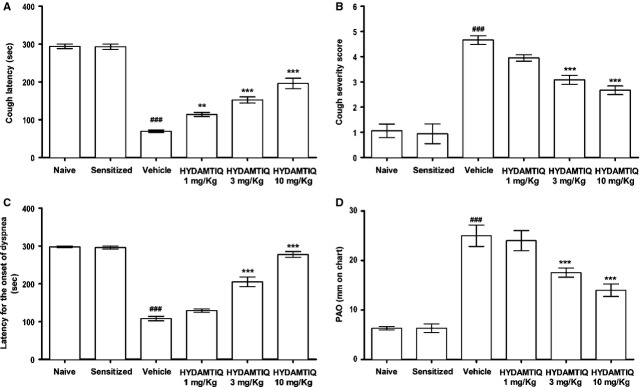
Respiratory signs in sensitized challenged guinea pigs. Evaluation of latency for the onset of cough (A), severity of cough (B), latency for the onset of dyspnoea (C), pressure of airway opening (PAO) after MeCh (200 μg/ml) aerosol (D). Data are means ± SEM. ^###^*P* < 0.001 *versus* Naive; **P < 0.01 and ***P < 0.001 *versus* Vehicle.

Sensitized guinea pigs, exposed for the second time to OVA aerosol, presented a series of striking abnormalities of the respiratory pattern, such as severe cough and dyspnoea. These signs started 1–2 min. after the beginning of antigen exposure. HYDAMTIQ treatment delayed the appearance and reduced the severity of cough (Fig. 2A–C). Clear-cut signs of dyspnoea were not detected in breath recordings obtained from animals treated with 3 or 10 mg/kg/day of the compound. Three days after OVA-challenge, airway hyper-responsiveness was tested with an aerosol of MeCh (200 μg/ml), maintained for 1 min. HYDAMTIQ reduced, in a dose-dependent manner, lung resistance to inflation (PAO; Fig. 2D).

### HYDAMTIQ treatment reduced lung histological changes caused by OVA-induced asthma

**Figure 3:**
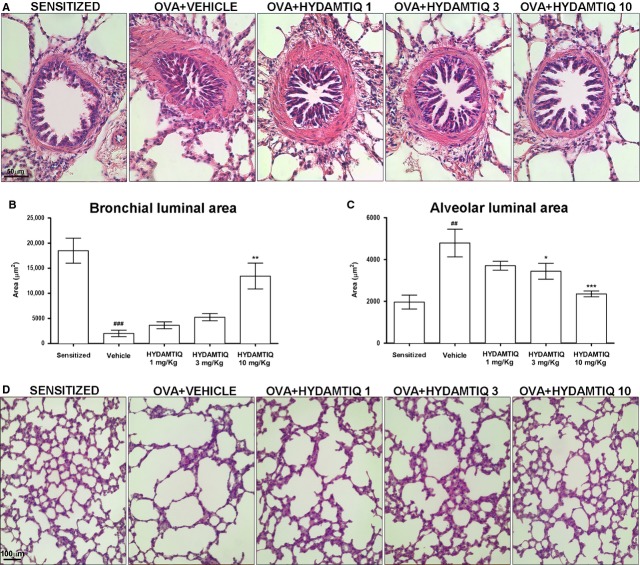
Evaluation of bronchiolar and alveolar luminal area in guinea pig lungs. The luminal area of bronchioles (A and B) and alveoli (C and D) were evaluated in haematoxylin-and eosin-stained sections and quantified by morphometric analyses. Data are means ± SEM. ^##^*P* < 0.01 and ^###^*P* < 0.001 *versus* Sensitized; *P < 0.05, **P < 0.01 and ***P < 0.001 *versus* Vehicle.

Histological examinations of lungs showed that the alveolar air spaces of OVA-sensitized not challenged animals were small sized and intrapulmonary bronchioles had open lumina with short folds of the mucosa, while OVA-challenged animals had markedly dilated respiratory air spaces and a reduction in the lumen of intrapulmonary bronchi, with long mucosal folds expanding in the bronchiolar lumen [13, 25]. The treatment with HYDAMTIQ at 3 and 10 mg/kg counteracted these respiratory abnormalities (Fig. 3A–D).

**Figure 4:**
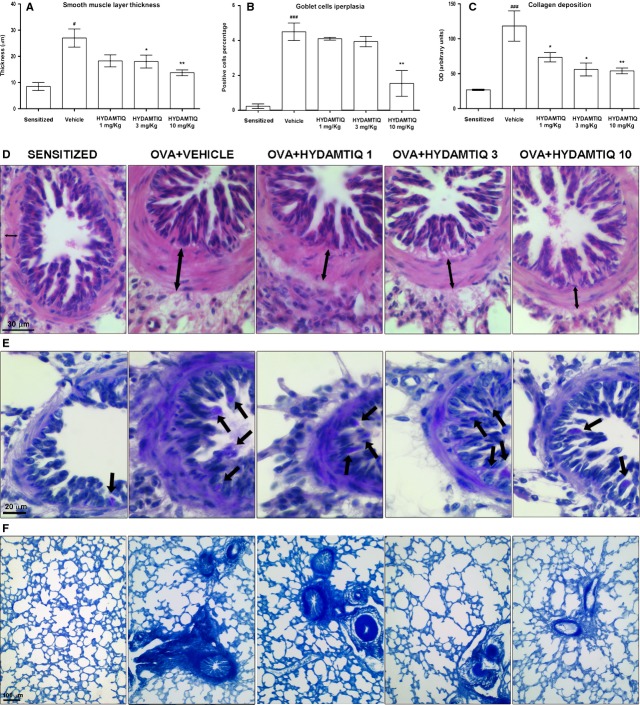
Histopathological evaluation of airway remodelling. (A and D) Smooth muscle layer thickness (see the arrows). (B and E) Goblet cells in PAS-stained lung sections (see the arrows). (C and F) Densitometric analysis of collagen deposition in Azan-stained lung sections. Data are means ± SEM. ^#^*P* < 0.05 and ^###^*P* < 0.001 *versus* Sensitized; *P < 0.05 and **P < 0.01 *versus* Vehicle.

We next studied if PARP inhibition could modify airway remodelling by evaluating the thickness of the smooth muscle layer and the relative number of goblet cells, which increases in response to airway insults with a resultant increase in the output of mucous [26]. As expected, the percentage of PAS-positive goblet cells over total bronchial epithelial cells, as well as the thickness of the airway smooth muscle layer, was significantly increased in the OVA-challenged animals in comparison to OVA-sensitized not challenged. HYDAMTIQ was able to reduce both markers of bronchial remodelling (Fig. 4A–D and B–E).

By evaluating the deposition of collagen, we studied the extent of pulmonary fibrosis, another crucial aspect of adverse remodelling, commonly observed in OVA-challenged guinea pigs. Figure 4C and F shows that HYDAMTIQ-treated animals had a decreased content of collagen in their lungs.

### HYDAMTIQ treatment reduced asthma-mediated inflammatory cell infiltration and oxidative stress damage in the lungs

**Figure 5:**
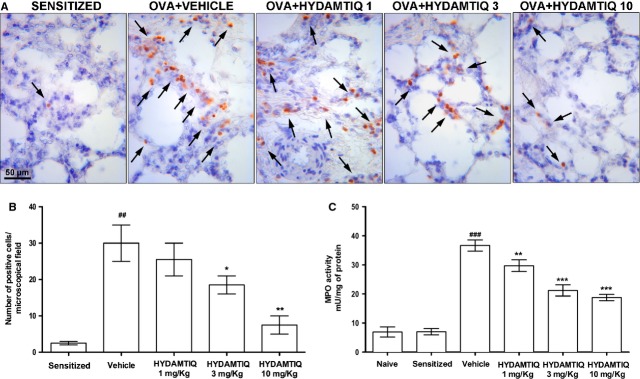
Evaluation of leucocyte lung infiltration. (A) Leucocytes are indicated by arrows. (B) Number of cells positive for eosinophilic major basic protein (eMBP). (C) Myeloperoxidase activity in the lung tissue. Values are means ± SEM. ^##^*P* < 0.01 and ^###^*P* < 0.001 *versus* Naïve or Sensitized; *P < 0.05, **P < 0.01 and ***P < 0.001 *versus* vehicle.

Myeloperoxidase activity, a marker of leucocyte infiltration into inflamed tissue, has previously been reported to significantly increase after OVA-induced asthma-like reaction in the lungs of sensitized guinea pigs [6, 13]. It has also been demonstrated that cells stained for eMBP are abundant in lung parenchyma of OVA-challenged guinea pigs [25]. Figure 5A–C respectively shows that HYDAMTIQ treatment significantly and dose-dependently reduced MBP-positive eosinophils and MPO activity in lungs of OVA-challenged animals.

**Figure 6:**
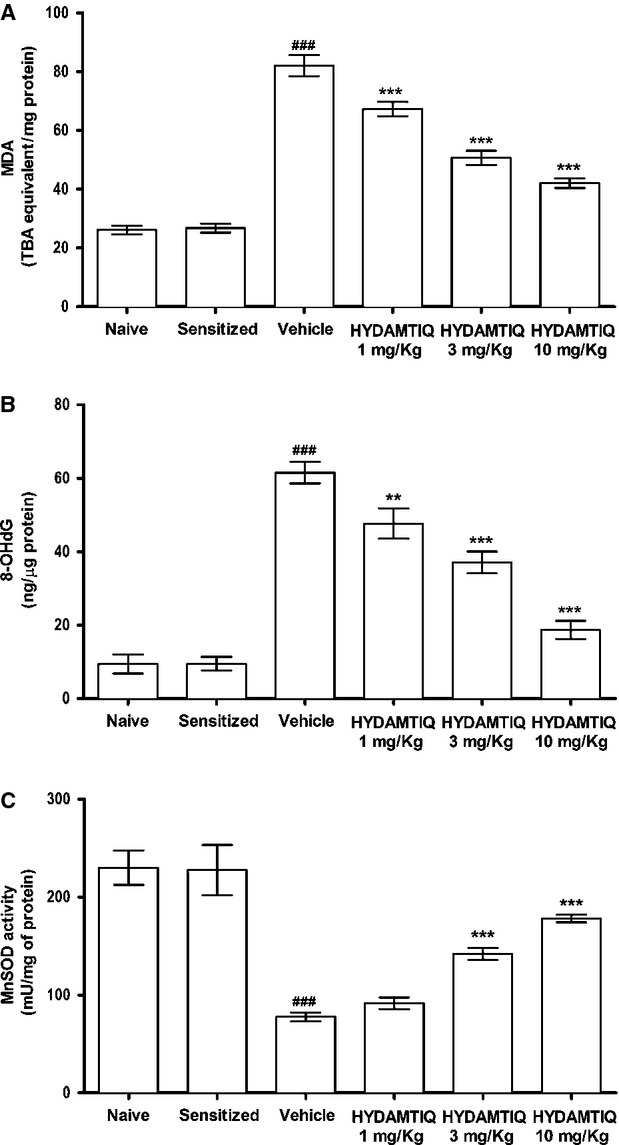
Evaluation of oxidative stress in lung tissue. (A) Levels of malondialdehyde, the end product of lipid peroxidation. (B) Levels of 8-OHdG, a marker of free radicals-induced DNA damage. (C) Activity of manganese superoxide dismutase (MnSOD), an enzyme rapidly inactivated by free radicals. Values are means ± SEM. ^###^*P* < 0.001 *versus* Naive; **P < 0.01 and ***P < 0.001 *versus* Vehicle.

Oxidative stress-induced damage of different cell types in the lungs after OVA-challenge has been repeatedly reported [7, 13, 25]. In the present study, we confirmed that OVA-induced asthma-like reaction causes an increased lung content of MDA, the end product of lipid peroxidation, and of 8-OHdG, a marker of DNA damage. Figure 6A and B shows HYDAMTIQ reduced this lung damage. The treatment also attenuated the allergen-induced decrease in MnSOD activity (Fig. 6C).

### HYDAMTIQ treatment reduced OVA-mediated histamine release from mast cells

**Figure 7:**
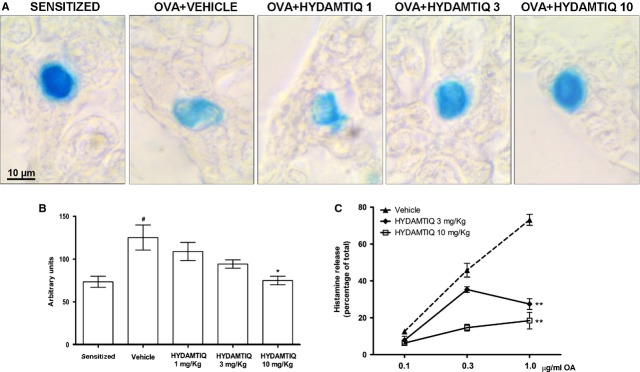
Antigen-induced mast cell activation. Representative micrographs of Astra blue-stained lung sections (A) and optical density (B) in sensitized or ovalbumin (OVA)-challenged animals treated with vehicle or HYDAMTIQ. (C) OVA-induced histamine release from purified mast cells obtained from the peritoneal cavity of sensitized guinea pigs. Values are means ± SEM. ^#^*P* < 0.05 *versus* Sensitized; *P < 0.05 and **P < 0.01 *versus* Vehicle.

A significant decrease in mast cells optical density upon staining with Astra blue, because of massive discharge of mast-cell granules, has been previously reported in the lungs of OVA-challenged, sensitized guinea pigs (Fig. 7A and B) [13]. HYDAMTIQ treatment attenuated mast-cell degranulation (Fig. 7B).

When isolated mast cells from peritoneal and pleural cavities of OVA-sensitized animals were exposed to different OVA concentrations, massive release of histamine (up to 75% of the total) occurred. Interestingly, mast cells obtained from HYDAMTIQ-treated animals, when exposed to the antigen *in vitro*, released a lower percentage of histamine that those obtained from vehicle-treated animals (Fig. 7C).

### HYDAMTIQ treatment reduced the increase of inflammatory cytokines in the lungs of OVA-exposed animals

It is generally accepted that asthma is associated with significant airway inflammation and with increased number of cells in BAL. We found that the total cell number in BAL obtained from OVA-challenged animals increased by approximately six times (from 1.6 × 10^6^ in sensitized to 7.4 × 10^6^ cells/ml in OVA-challenged animals; *P* < 0.01).

Other inflammatory markers evaluated in the lungs were also changed and Table 1 shows that HYDAMTIQ treatment attenuated the challenge-induced increase of TNFα, IL-1β, IL-5, IL-6 and IL-18 in the supernatant of guinea pig lung homogenates.

**Table 1:** Cytokine content in lungs or BAL

	PGD_2_† (ng/μg)	TNF-α (ng/μg)	IL-1β (pg/μg)	IL-5 (pg/μg)	IL-6 (ng/μg)	IL-18 (pg/μg)
Naive	5.6 ± 0.2	6.6 ± 0.6	1.5 ± 0.06	3.9 ± 0.3	3.6 ± 0.1	1.9 ± 0.2
Sensitized	5.8 ± 0.3	6.5 ± 0.4	1.7 ± 0.05	–	–	–
Vehicle	34.1 ± 2.6*	24.7 ± 2.2*	6.7 ± 0.4*	4.9 ± 0.2*	9.3 ± 0.7*	11.2 ± 0.5*
HYDAMTIQ 1 mg/Kg	22 ± 2.2**	21.1 ± 1.9	5.2 ± 1.3**	3.4 ± 0.2**	6.4 ± 0.7**	8.4 ± 0.3**
HYDAMTIQ 3 mg/Kg	20.1 ± 1.3***	15.5 ± 1.7***	4.9 ± 0.5**	3.5 ± 0.07**	7.5 ± 1.1**	8.4 ± 0.6**
HYDAMTIQ 10 mg/Kg	8.3 ± 0.6***	11.1 ± 0.6***	2.8 ± 0.2***	1.8 ± 0.1***	5.1 ± 0.3***	4.9 ± 0.6***

† PGD_2_ was measured in BAL supernatant. TNFα, IL-1β, IL-5, IL-6 and IL-18 in the supernatant of lung homogenates. “-”: not determined. The values are expressed as ng or pg of protein/μg of total proteins. Values are means ± SEM.

*** *P* < 0.01 and

** *P* < 0.05 *versus* Vehicle

* *P* < 0.01 *versus* Naive.

Finally, to monitor the extent of mast-cell degranulation *in vivo*, we evaluated the concentrations of PGD_2_ in BAL supernatants of sensitized, OVA-challenged guinea pigs and we found that HYDAMTIQ treatment reduced PGD_2_ concentrations, suggesting that treated animals had reduced mast-cell activation (Table 1).

## Discussion

HYDAMTIQ, a new potent and selective PARP inhibitor, has been previously shown to significantly improve the long-term neurological outcome in rats with middle cerebral artery occlusion by significantly reducing brain infarct volumes and leucocyte infiltration [24]. In view of the importance of leucocyte recruitment, inflammatory damage and the previously observed positive actions of first generation PARP inhibitors in asthma models [6, 12], we studied the effects of repeated HYDAMTIQ administration on the functional, biochemical and morphological lung changes induced by OVA exposure in sensitized guinea pigs. The results here reported show that HYDAMTIQ did reduce not only cough, dyspnoea and the increase of PAO, which is an indication of acute bronchospasm, but also the delayed airway hypersensitivity to inhaled MeCh. It also reduced intrapulmonary bronchial obstruction, alveolar changes, smooth muscle hyperplasia and lung infiltration of eosinophils or other inflammatory cells. The asthma model we used is generally considered a suitable model to evaluate agents and tools that may be helpful for the clinical treatment of this disease [38] and it has been in use in our laboratory for several years [6, 13, 25, 26, 28, 39–42].

The molecular mechanisms linking PARP inhibition to the reduced signs of asthma-like reaction and the attenuation of the airway inflammatory and remodelling events after OVA-challenge have not been clarified in details. Interestingly, however, mast cells obtained from sensitized, HYDAMTIQ-treated animals, released a reduced percentage of their histamine when exposed to antigen *in vitro*. This may suggest that the changes in the processes leading to activation of sensitized mast cells as a consequence of a repeated antigen exposure contribute to the overall HYDAMTIQ action. A number of other cellular and molecular mechanisms may however contribute to the overall HYDAMTIQ action in this model [11, 20, 21].

Most cell types present in the lungs express abundant PARP-1 and PARP-2 in their nuclei and PARP inhibitors can modify their sensitivity to oxidative stress and possibly their role in asthma pathophysiology. It is indeed widely accepted that PARP-1 *knock-out* mice or the treatment with PARP inhibitors may decrease oxidative stress-induced tissue damage in most of the organs so far studied [43]. As an increase in reactive oxygen species both in experimental and human asthmatic disorders has been widely shown [44], we suggest that HYDAMTIQ administration could be a promising new pharmacological tool to reduce the pulmonary damage induced by acute asthma-like reaction. In the present study, we confirmed this working hypothesis and showed that repeated HYDAMTIQ administration attenuated the oxidative stress damage in the lungs: the treatment indeed reduced the level of markers of free radicals-induced injury to membranes (MDA), nuclei (8-OHdG) and mitochondria (MnSOD; Fig. 6). As mentioned above, DNA damage activates PARP-1 [45] and causes NAD and ATP cell depletion and, eventually cell death [14, 46]. Poly(ADP-ribose) polymerase activation may also lead to release of AIF from the mitochondria, facilitates AIF translocation to the nucleus and activation of a caspase-independent programmed cell death which can cause loss of lung endothelium and airway wall [15]. The reduction in oxidative lung damage after an acute asthma attack could therefore represent one of the mechanisms of the overall HYDAMTIQ therapeutic action. It has also been demonstrated that PARP activation promotes the expression of P-selectin and ICAM-1 in lung endothelial cells [12] and as endothelia activation can regulate leucocyte chemo-attraction, adhesion and migration into the tissue upon allergen challenge, it is reasonable to assume that the reduction in MPO activity and the reduced number of eMBP positive eosinophils found in the lungs of HYDAMTIQ-treated animals (Fig. 5) could be mediated by the reduced expression of the above mentioned adhesion molecules by vascular endothelium. Furthermore, PARP-1 and PARP-2 are key regulatory enzymes of immune cell function and promote the expression of inflammatory genes in macrophages [16, 47], granulocytes [48], T and B lymphocytes [49] possibly because they contribute to the activation of transcription factors such as NF-kB and AP-1 [7, 16–19]. Finally, asthma-induced damage of lung endothelial cells can cause excessive exposure of DC to airborne antigens and infectious agents. Previous studies from our and other laboratories have shown that DC exposed to PARP inhibitors display a reduced expression of the pro-inflammatory cytokine IL-12, of the co-stimulatory antigen CD86 [22] and a decreased ability to promote T-cell proliferation [23]. It is therefore not surprising that PARP inhibitors significantly attenuate lung inflammation and remodelling after acute asthma attacks. A limitation of this study consists in the fact that the model is not designed to specifically evaluate all the aspects of airway remodelling, a phenomenon occurring in the long term upon chronic exposure to allergens. This study highlights the initial phase of the remodelling process, considering that the guinea pigs underwent two OVA challenges during the second and third week after sensitization and MeCh aerosol 3 days before tissue sampling.

In conclusion, the results here reported are in line with the suggestion that PARP inhibitors can have a therapeutic potential in the treatment of severe asthma-induced lung inflammation, remodelling and fibrosis.
